# Discovering Transcription and Splicing Networks in Myelodysplastic Syndromes

**DOI:** 10.1371/journal.pone.0079118

**Published:** 2013-11-14

**Authors:** Hongyan Wang, Jianguo Wen, Chung-che Chang, Xiaobo Zhou

**Affiliations:** 1 Center for Bioinformatics and Systems Biology, Division of Radiologic Sciences, Wake Forest University Baptist Medical Center, Winston-Salem, North Carolina, United States of America; 2 Department of Pathology, the Methodist Hospital Research Institute, Houston, Texas, United States of America; 3 Department of Pathology, University of Central Florida, Orlando, Florida, United States of America; UT MD Anderson Cancer Center, United States of America

## Abstract

More and more transcription factors and their motifs have been reported and linked to specific gene expression levels. However, focusing only on transcription is not sufficient for mechanism research. Most genes, especially in eukaryotes, are alternatively spliced to different isoforms. Some of these isoforms increase the biodiversity of proteins. From this viewpoint, transcription and splicing are two of important mechanisms to modulate expression levels of isoforms. To integrate these two kinds of regulation, we built a linear regression model to select a subset of transcription factors and splicing factors for each co-expressed isoforms using least-angle regression approach. Then, we applied this method to investigate the mechanism of myelodysplastic syndromes (MDS), a precursor lesion of acute myeloid leukemia. Results suggested that expression levels of most isoforms were regulated by a set of selected regulatory factors. Some of the detected factors, such as EGR1 and STAT family, are highly correlated with progression of MDS. We discovered that the splicing factor SRSF11 experienced alternative splicing switch, and in turn induced different amino acid sequences between MDS and controls. This splicing switch causes two different splicing mechanisms. Polymerase Chain Reaction experiments also confirmed that one of its isoforms was over-expressed in MDS. We analyzed the regulatory networks constructed from the co-expressed isoforms and their regulatory factors in MDS. Many of these networks were enriched in the herpes simplex infection pathway which involves many splicing factors, and pathways in cancers and acute or chronic myeloid leukemia.

## Introduction

Gene expression levels are highly dependent on the regulation of transcription factors which mainly bind to the near-promoter regions to facilitate or block the recruitment of DNA polymerase II (pol II) and other complexes. Some methods have been proposed to predict - gene expression using such binding information of transcription factors [1], [2]. Conlon *et al*. [2]suggested associating gene expression with the interaction strength between the upper stream of the gene and motifs of its transcription factors. This interaction was defined in terms of degree of matching and occurrences of binding sites. After that, a probabilistic approach was proposed to infer regulatory rules of transcriptional networks from gene expression data and DNA sequences [1]. This method started with finding co-expressed genes, and then extracted a large number of putative regulatory DNA motifs in these co-expressed genes. However, these methods only considered transcription levels extracted from gene array data and lost sight of other mechanisms such as alternative splicing. Moreover, these methods may suffer high false positive rate when mining transcription factor binding sites. To control the rate of false positive, integrating information from other kinds of data, like conservation data, is also necessary.

Alternative splicing is one of the most versatile mechanisms of gene expression regulation and accounts for a considerable proportion of proteomic complexity in higher eukaryotes. The mRNA isoforms produced by this alternative processing comprise of different combination of exons, and may differ in structure, function, and other properties [3], [4]. Different mRNA isoforms are translated into different protein isoforms (if they exist) which may have related, distinct or even opposing functions.

The alternative splicing process is regulated by many types of RNA binding proteins, especially the heterogeneous nuclear ribonucleoproteins (hnRNP) and the serine/arginine-rich (SR) family. In eukaryotic cells, hnRNP proteins participate in almost all pre-mRNA processing steps including splicing, mature mRNA export, localization, translation, and stability [5], [6]. SR proteins are involved in regulating and selecting splice sites in eukaryotic mRNAs. These proteins, either ‘classical’ SR proteins or additional SR proteins [7], generally have at least one RRM (RNA recognition motif) domain for RNA binding and a C-terminus RS domain for controlling interactions with other proteins (including other SR proteins). In addition to alternative splicing, the SR proteins are also involved in mRNA nuclear export and mRNA translation. One of the most important characteristics of these splicing factors is their functional specificity. Many animal experiments suggest that the RNA binding ability of individual SR proteins are sequence-specific and their ability to regulate alternative splicing is different [8]–[10].

These highly specific and non-redundant characteristics of splicing factors motivated researchers to look for association between abnormalities in SR proteins and the development of human cancers. Although the underlying mechanisms are elusive and need further studies, splicing factors that regulate specific pathways in diseases can be treated as putative markers, especially when their targets in these disease-related pathways have experienced alternative splicing and produced different protein isoforms.

In this study, we built a systematic method to identify transcription factors and splicing factors that regulated genes to produce different RNA isoforms in diseases. Our framework consists of four steps. First, differentially expressed mRNA isoforms (DEIs) are extracted by comparing abnormal cells and controls using RNA-seq analysis tools [11]. These DEIs that may experience abnormal splicing are putative targets of splicing factors that might function abnormally in disease. Assuming that co-expressed genes have a good probability of being functionally related, we clustered these DEIs to co-expressed groups using hierarchical clustering method. These co-expressed isoforms may be regulated by the same group of transcription factors (TFs) and splicing factors (SFs). Since co-expressed mRNAs are more likely to have their promoter regions bound by common transcription factors, we constructed a dataset called promoter region dataset (PRD) to mine the TF-isoform interactions (for example, co-expressed isoforms may also have common splicing factors that bind to the regions near their splicing sites). We then constructed an exon-intron (centered at splicing sites and extending 200 bp on both sides) dataset (EID) to explore SF-isoform interactions. The binding strength of the TF-isoform interactions is quantified by scoring the transcription factor’s binding sites in the promoter region. Similarly, the binding strength of the SF-isoform interactions is defined by scoring the splicing factor’s binding sites in the exon-intron regions of pre-mRNA. To integrate both kinds of regulation, we built a linear regression model and selected a subset of transcription factors and splicing factors that can regulate the expression of co-expressed isoforms using least-angle regression (LARS) [12] selection approach.

The proposed method was applied to a RNA-seq dataset comprising of 4 myelodysplastic syndromes (MDS) samples (RAEB subtype) and 5 matched controls. MDSs are defined as clonal stem cell disorder characterized by ineffective hematopoiesis and impaired difference in some bone marrow lineages, leading to peripheral-blood cytopenias. According to the WHO [13], the main categories of MDS include refractory cytopenia with unilineage dysplasia (RA), refractory cytopenia with ringed sideroblast (RARS), refractory cytopenia with multi-lineage dysplasia (RCMD), refractory cytopenia with excess blasts (RAEB), 5q-syndrome and unclassifiable MDS. In this paper, we focused on RAEB cases which are characterized by 5–20% myeloblasts in the marrow [14]. This is a higher risk subtype and likely to transform to acute myelogenous leukemia (AML).

Some work studied the genetic abnormality of MDS with mutations of key genes [15], e.g. TP53 and RUNX, which are highly related with poor overall survival. Recently, the recurrent mutation of splicing factor U2AF1 has been validated for MDS patients [16]. In-vitro experiments demonstrated that the mutated U2AF2 enhances splicing and promotes exon skipping. These genetic aberrations would improve the prediction of prognosis and development of novel treatment. However, mutation detection cannot determine which genes are altered due to the abnormality of splicing factors. Our method can recognize not only isoforms that experience an abnormal splicing process but also their putative regulatory factors. The co-expressed isoforms and their transcription and inferred splicing factors comprise our transcription and splicing networks.

To verify the biological significance of these regulatory networks composed of a group of co-expressed isoforms and their regulatory factors in MDS, we showed theses networks were significantly enriched (*p*<0.0001) in some known Gene Ontology (GO) biology processes and Kyoto Encyclopedia of Genes and Genomes (KEGG) pathways. Enrichment analysis demonstrated that more than half of these networks were enriched in herpes simplex infection due to involving many splicing factors. Six networks were significantly related with pathways in cancer, and five networks were significantly related with acute or chronic myeloid leukemia (AML).

## Methods

**Figure 1:**
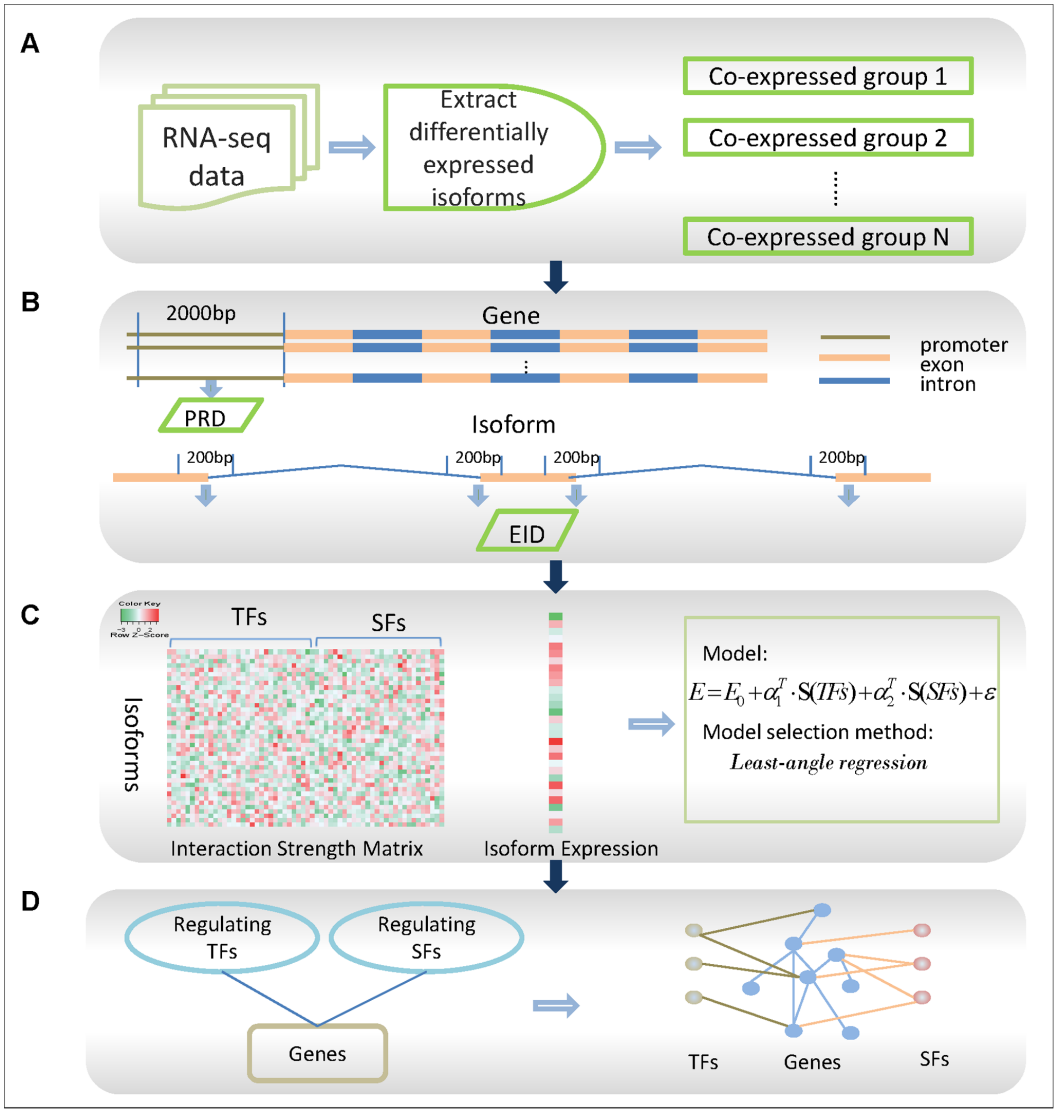
Flowchart of proposed method for constructing regulatory networks. Flowchart of proposed method for constructing regulatory networks: (A). Process raw RNA-seq data, find out deferentially expressed isoforms using Tophat and Cufflinks and cluster these isoforms to get gene cluster that may be regulated by same TFs and SFs. (B). Construct two dataset, promote region data (PRD) and exon-intron data (EID), for mining the interaction strength of the TF-isoform interactions and SF-isoform interaction. (C). Use interaction strength to predict the expression levels of isoforms in a co-expressed group. (D). Link model-selected TFs and SFs with their target genes.

Our four-step framework (**Figure 1**) starts with raw RNA sequencing (RNA-seq) data. First, differentially expressed isoforms (DEIs) are identified from a large amount of isoforms. We cluster these DEIs into co-expressed groups that may be regulated by common TFs and SFs (**Figure 1A**). At the second step, two datasets, promoter region data (PRD) and exon-intron data (EID), are constructed, where the PRD dataset is used for mining the interaction strength between TFs and isoforms and the EID is used for exploring the interaction strength between TFs and isoforms (**Figure 1B**). This step outputs two interaction strength matrices with rows corresponding to isoforms and columns corresponding to TFs or SFs. Then, by taking the interaction strength matrices as observations of explanatory variables, we build linear model to regress the expression levels of isoforms in a co-expressed group. To detect the most important factors that regulate a co-expressed group, a reliable model selection method called least-angle regression (LARS) is applied (**Figure 1C**). At the final step, the TFs and SFs selected by the LARS are linked with their target genes, forming regulatory networks (**Figure 1D**).

### Ethics Statement

This study was approved by the Institutional Review Board of The Methodist Hospital, Houston, Texas, USA and the need for written informed consent from the participants was waived by the IRB.

### Data Preprocessing and Co-expressed Isoforms

RNA samples were prepared from 9 individuals, 4 samples from RAEB patients and 5 controls (http://ctsb.is.wfubmc.edu/MDS/MDS.html). After sequencing using Genome Analyzer II (GAII) (Illumina, San Diego, CA), millions of reads were produced for each sample. The raw data were processed using a RNA-seq analysis pipeline [11]. Briefly, reads in the FASTA format were first aligned to whole genome using TopHat software. Since our sequencing data are pair-ended, the fragments were selected at 350 bp and the length of reads was 76, the inner distance between mate pairs was set as 198. The standard deviation for the distribution on inner distances between mate pairs was set as 30 based on our estimation. Then Cufflinks was called to estimate the expression level (RPKM, reads per kilobase of transcript per million mapped reads) of each isoform. Then, Cuffdiff was used to identify differentially expressed isoforms (using default parameters). This protocol returned 1056 isoforms that were differentially expressed in MDS compared with controls. Before using these DEIs to build splicing networks, we first filtered out those isoforms that had not been validated at the protein level. Then, we checked the condition (control or MDS) in which an isoform was up-regulated and required that all FPKM values under this condition were higher than 5 [17].

Although there are debates on whether co-expressed genes are functionally related, numerous studies have suggested that at least some co-expressed clusters function together [18]–[20]. Here we applied hierarchical cluster analysis to those differentially expressed isoforms to obtain co-expressed groups. The distances between genes were defined as Pearson correlation. Allocco *et al.* [21] concluded that genes with strongly co-expressed mRNAs were more likely to have their promoter regions bound by common transcription factors. However, this co-regulating effect was significant only when expression of these co-expressed mRNAs were highly correlated. To control the similarity level of expression profiles of isoforms in the same group, we limited the size of each group to be between 10 and 30 [19], [22]. In our MDS study, this resulted in 34 clusters (**Table 1** **)**.

**Table 1:** Regulatory networks found by our model.

No	Target Genes	Transcription Factors	Splicing Factors
**1**	GNB1, GPATCH4, MRPL51, BRD4, ODC1, ATP5J, HNRNPD, RBBP7, CALM3, ANP32C, PSPC1, SETD3	Blimp-1, FOX factors, FOXP1a, Sp1, STAT5B	MBNL1, PSF, Sam68, SF2, SRp38, YB-1
**2**	PARK7, KDM1A, SSR2, DNTT, HNRNPA1L2, SUPT16H, SLIRP, YWHAE, TAF15, TOP2A, SRSF1, ATP5A1, EEF1B2, HMGN1, U2SURP, HSD17B4, CANX, MAP7D3, HNRNPM, BCL11A	Egr-1, Evi-1, LXR direct repeat 4, MEF-2, Nkx2-5, Pax-6, RSRFC4, Sp1	ETR-3, hnRNPD, hnRNPI(PTB), hnRNPQ, MBNL1, PSF, SF2, SRp38, SRp40, hnRNPC/C1/C2
**3**	TARDBP, HDAC1, ARHGDIB, ESD, CX-CR4, TTC1, HLA-DPB1, ANP32B, ATP1A1, ARPC5	ATF6, COUPTF, FOX factors, FOXP1d, IRF, Nkx3-1	SRp40, hnRNPE1/E2
**4**	PRDM2,GPBP1L1,ZRANB2,DNTTIP2,SMG7,EIF4G2,PHF21A,RBM4B,GNPTAB,UFM1,ERCC5,SF3B1,CD47,CDV3,BDP1,DDAH2,SNX3,PPIL4,KIT,PCMTD1,RABGAP1,DDX3X,C16orf80	Blimp-1, Egr-1, Evi-1, FOX factors, KROX, NIT2, Pax-6, POU3F2, RSRFC4, SRF	hnRNPQ, KSRP, SF1, SRp38, SRp54, SRp55, TIA-1, TIAL1, YB-1, hnRNPA1/A2, hnRNPE1/E2
**6**	SRRM1, JUN, PRDX6, DUSP10, RBM34, HSP90AA1, SRRM2, RPS7, RPL3, RPL32, RPS20	Blimp-1, HNF-1alpha, KROX, MEF-2, Nkx2-5, Sp1	SRp20, SRp54, YB-1
**7**	TMEM50A, DNAJC8, ZNHIT6, MRPL9, DDX21, RPL36, RPL13A, NOP58, YWHAB, SREK1, ZMAT2, EIF2AK1, RBM3, UPF3B, HMGB2, SRF	Blimp-1, Egr-1, ER-alpha, Evi-1, LXR direct repeat 4, Nkx2-5, Pax-6, Pbx1a, PLZF	SC35, SF2, SRp40, TIA-1, hnRNPA1/A2
**8**	STMN1, ATPIF1, CFL1, APLP2, SARNP, MLEC, POMP, ERH, FUS, NME1, GNAI2, HNRNPAB, DEK, HIST1H2BC, HIST1H2AC, HIST1H4H, SEC61G, HIST1H2BK	AP-2alphaA, Egr-1, HOXA5 (Hox-1.3), MCM1, MTF-1, NF-Y, Pax-6, PLZF, RXR-alpha, STAT1	hnRNPF, hnRNPI(PTB), MBNL1, SRp30c, SRp55, TIAL1
**9**	SRSF4, NASP, SERBP1, CSTF3, SSRP1, ERP29, RAN, HMGB1, SRP14, PSMA4, NUTF2, RPS16, VAMP8, HSPE1, CCT5, TAF9, HIST1H2BD, HIST1H4E, TUBB, HNRNPA2B1, PPIA, FKBP3	AP-2alphaA, Egr-1, ER-alpha, Evi-1, GLI1, IRF, MCM1, NIT2, Nkx2-5, Pbx1a, RSRFC4, STAT1, STAT5A (homotetramer), UF1H3BETA	9G8, hnRNPI(PTB), HTra2alpha, SRp30c, TIAL1
**10**	KHDRBS1, PIP4K2A, RPLP2, SMARCC2, RBM25, RFX7, YWHAQ, XRCC5, VDAC1, GNB2L1, SHFM1, SMC1A, HDGF, LSM14A, MAT2A	AP-2alphaA, ATF6, Egr-1, MTF-1, Nkx2-5, STAT5B, ZID	9G8, HTra2alpha, MBNL1, PSF, SC35, SRp30c
**11**	SFPQ, SRSF11, BCAS2, TFAM, ZFP91, CSDA, NGRN, RPS15A, RPL26, NACA2, EEF2, DDX18, SF3B1, MAPRE1, SATB1, RPL24, SMARCA5, RUFY1, NUP153, DDX39B, RPL10A, HOXA7, RPL7, TCEAL4, RBMX, SYF2	ER-alpha, GLI1, KAISO, mat1-Mc, MIG1, MTF-1, Nkx2-5, Pax-6, PLZF, RSRFC4, Sp1, SRF, STAT1, ZID	9G8, DAZAP1, hnRNPF, HTra2beta1, KSRP, MBNL1, SRp30c, TIA-1, hnRNPC/C1/C2, hnRNPE1/E2
**12**	THRAP3, PSMB4, ARF3, TCF25, GABARAP, TAF15, CHMP2A, GAR1, C9orf78, ACIN1, UBB, BASP1	Egr-1, Gfi1b, NF-Y, STAT5B, UF1H3BETA	PSF, SC35, SRp40, SRp55
**13**	GNL2, EIF3M, CD44, FNBP4, OSBPL8, NUFIP2, STK17B, SYPL1, MDM4, ANKRD36B, IER2	FOXO3a, POU3F2, TBX5	9G8, ETR-3, hnRNPF, hnRNPP(TLS), SRp20, hnRNPH1/H2/H3
**14**	NASP, UQCRH, ZRANB2, NUCKS1, FKBP4, ANP32A, EIF3C, PTMA, SUMO3, HNRNPA2B1, TXN, MYL12B	AIRE, COUP-TF1, Egr-1, Evi-1, GLI1, MTF-1, NF-kappaB, STAT5A (homodimer), UF1H3BETA	Sam68
**15**	EPS15, RAB13, ERLIN1, CAT, CWC15, RPL6, EID1, SEC11A, CKLF, RPL23, RPL19, FBXO7, EIF3L, POLR2B, RPL34, RPS3A, RPS6, CNTRL, EIF2S3, IK, HMGN4	AIRE, COUPTF, Egr-1, Evi-1, FOXP1a, HNF-1alpha, LXR direct repeat 4, Nkx2-2, Nkx2-5, PLZF, Sp1, SRF, PPARalpha:RXRalpha, PPARG	hnRNPP(TLS), KSRP, MBNL1, SF2, SRp55
**16**	FUBP1, UFC1, HNRNPU, CHD4, ETV6, FGFR1OP2, PFDN5, NCKAP1L, NACA, NAP1L1, CTCF, GLOD4, ZNF830, RPL38, SAFB2, CEBPZ, CEP63, USO1, HNRNPD, PPWD1, NSA2, RPS23, HINT1, SKP1, RPS12, EIF3H, CEP350, ARID4A, PRPF40A, ST13, STXBP3, SERTAD2, CNBP	AIRE, AP-2, ATF6, Blimp-1, Egr-1, Evi-1, KROX, LXR direct repeat 4, MEF-2, Nkx2-5, 1-Oct, POU3F2, RSRFC4, Sp1, SRF, ZBRK1	9G8, hnRNPK, hnRNPP(TLS), hnRNPQ, hnRNPU, SRp20, SRp30c, SRp38, SRp40, TIA-1, YB-1, hnRNPA1/A2, hnRNPC/C1/C2, hnRNPE1/E2
**17**	PHTF1, H3F3C, TRIAP1, PMAIP1, KLF2, EEF1B2, IL8, HNRPDL, ARRDC3, FKBP5, NAMPT	HNF4, HSF1, MafA, MIG1, PLZF, Sp1, ZID	9G8, KSRP
**18**	MAN1A2, IFI16, UPF2, FGFR1OP2, FLT3, TERF2IP, LRRFIP1, EGR1, MLL5, TMEM66, IRF2BP2, ITSN2, PRRC2C, STT3B, CD164, SPRY1	Egr-1, Nkx2-5, p53, RSRFC4, SRF, STAT1, STAT5B (homodimer), UF1H3BETA	9G8, MBNL1, TIA-1, hnRNPA1/A2, hnRNPC/C1/C2, hnRNPH1/H2/H3
**19**	HIST2H2BE, SLC2A3, SRSF5, TEC, PTP4A1, ESYT2, LYN, SMARCA2, JMJD1C, PIM3, KLF10	Egr-1, Nkx2-5, Pbx1a, Sp1	PSF
**20**	ANP32E, ILF2, COX4I1, AATF, MPO, SNRPD1, FBL, RPS19, SNRPD2, RPL18, PPM1G, RPS21, GCFC1, CCDC72, H2AFZ, COX6C, DKC1	LXR direct repeat 4, Nkx2-5, 1-Oct, PPARalpha:RXRalpha, REST, STAT5A (homodimer), STAT5B, TBX5, UF1H3BETA	hnRNPD, hnRNPU, HTra2alpha, SRp40, SRp55
**21**	GATAD2B, GDI2, APBB1IP, RPS13, HNRNPA1, LTA4H, MFAP1, ME2, MYADM, SEPT2, C21orf59, RUNX1, RPL35A, SNX2, HSPA9, PAIP2, LARS, SOX4, POLR2K, STRBP, RPL7A, SLC25A5, NRD1, LARP1, C19orf77, MRPL3, PNRC2, MTIF3	ATF6, Egr-1, ER-alpha, HNF-1alpha, IRF-1, MEF-2, MTF-1, Nkx2-5, PLZF, RSRFC4, Sp1, Sp3, UF1H3BETA, ZID	ETR-3, MBNL1, SRp38, SRp54, SRp55, TIAL1, hnRNPA1/A2, hnRNPE1/E2
**22**	SELL, TMEM123, DDX6, TMBIM6, CASC4, B2M, CHD2, USP34, SETD2, PAK2, HSD17B11, GOLPH3, DFIP1, WRNIP1, UTRN, VAMP2, PCM1, SORL1, PAN3, ATXN3, XRN2, FBXO11, C4orf3, GAPT	E2F-1:DP-2, Egr-1, GLI1, HNF-1alpha, IRF, IRF-3, KROX, mat1-Mc, MEF-2, MyoD, NF-kappaB, NIT2, Nkx2-5, NRSF, RSRFC4, Sp1, ZID	hnRNPI(PTB), KSRP, SF2, SRp20, SRp30c
**23**	RGS1, PTMS, PARP2, C16orf61, CLK1, EAF2, SLU7, RSRC2, ZCCHC7	AIRE, Evi-1, FOXO3a, IRF-3, MEF-2, VDR, CAR, PXR	hnRNPD
**24**	RGS2, CD34, RSF1, WNK1, CBFB, CSTB, IQGAP2, GNAI1, LAPTM4B, SH3KBP1, HNRNPM, ARCN1, MARCH6	AP-2, BLIMP1, KROX, MEF-2, Sp3, STAT5B	Sam68, hnRNPA1/A2
**25**	HNRNPU, SAP18, RBM26, LEO1, UQCRC2, RPL27, RPS9, DUSP11, DARS, ARPC2, ESF1, SERINC3, PITPNB, HNRNPA0, TCERG1, NPM1, C6orf48, IL2RG, RPS4X, API5, SEP15, RPL23A	Blimp-1, Egr-1, Evi-1, GLI1, MTF-1, NIT2, Nkx2-2, Pax-6, PLZF, ZID	DAZAP1, hnRNPD, hnRNPI(PTB), MBNL1, SRp20, SRp30c, YB-1, hnRNPA1/A2, hnRNPE1/E2
**27**	EIF4EBP2, CELF1, ATXN7L3B, PRPF8, RPSA, SRP72, DDX46, STK38, SRSF3, NGFRAP1, TOP2B	BLIMP1, COUP-TF1, Egr-1, HNF-1alpha, IRF, IRF-1, SRF, ZID	YB-1
**28**	PDLIM1, LMO2, C11orf67, SLC38A1, DUSP6, ROCK2, KIF2A, PHF3, CD164, TAX1BP1, SEPT6, DDX1	Egr-1, HNF-1alpha, RXR-alpha	ETR-3, hnRNPK, SRp55
**30**	PSMA1, NEDD8, ADAM10, PSMC5, UBE2G2, PDCD10, SEC62, CWC27, LCP2, XPO7, PEBP1	AIRE, ATF6, FOXO1, Gfi1b, 1-Oct, Sp1	hnRNPF, SRp20, TIA-1
**31**	NXF1, FAU, TAF1D, PPTC7, PABPC3, ELF1, CHD9, JUND, LAPTM4A, RANBP2, HAT1, UBE2D3, SBDS, FLJ44635, TSC22D3, HSPH1, KPNB1	ER-alpha, ER-beta, GLI1, KROX, Nkx2-5, NRSF, Staf	HTra2alpha, Sam68, SRp30c, SRp38, SRp55, hnRNPA1/A2
**32**	CCND2, METAP2, ARGLU1, MIS18BP1, CCNK, PSME3, RPL38, GLTSCR2, RPS11, HADHA, RPL31, HSPD1, RPL37A, NOP56, BRK1, RPL37, UBE2V2	AIRE, AP-2alphaA, dl, Egr-1, HNF-1alpha-A, 1-Oct, Olf-1, p53, Pax-6, Pbx1a, RSRFC4, Sp1	SC35, SRp38, hnRNPE1/E2
**33**	ITM2B, SCAPER, PPIG, MRFAP1L1, RASGEF1B, AIF1, RNPC3, H3F3B, BPTF, AFF1, B3GNT5	Alx-4, Blimp-1, BLIMP1, HNF-1alpha, KROX, Sp1	hnRNPF, SRp40, hnRNPA1/A2
**34**	NIN, ZNF609, ZFP36L2, SOD1, NOL7, LTB, ITGA6, WDR77, PIM1, SEPT7	ABF, Antp, COUP-TF1, ID1, KAISO, myogenin/NF-1, RORalpha1	hnRNPK

The first column is differentially expressed isoform groups in our cases, the second and third columns are the Transcription factors and splicing factors predicted by our regression model. Some cells are blank, which means no corresponding factors for that co-expressed group.

### Mathematic Modeling

Transcription factors regulate transcription, which controls gene expression. Splicing factors regulate alternative splicing, which splices pre-mRNA to RNA isoforms and in turn changes protein expressions [23]. Some predictive methods have modeled gene expression levels as a linear function of occurrences of TF-binding sites (TFBSs) [2], [24]. In this paper, we focus on RNA isoforms instead of genes and try to link isoform expression levels not only with the transcriptional factors but also with splicing factors. Isoform expression levels were formulated as linear regression of the strength of TF-isoform interactions and SF-isoform interactions, given by
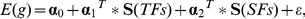
(1)where, 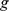 is an isoform; 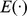 denotes the expression level; 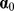, 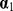 and s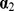 are regression coefficients; 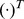 is transposition operator; 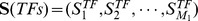 are binding strength vectors of transcription factors; 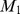 is the number of transcription factors; 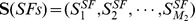 are binding strength vectors of splicing factors; 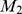 is number of splicing factors and 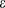 is the error term.

To estimate the binding strength between TFs and isoforms, we first constructed a promoter region database (PRD, **Figure 1B**) comprised of promoter sequences of isoforms. As in previous study [25], we extracted 2000 bp upstream of the transcription start sites as promoter regions. Similarly, we constructed an exon-intron dataset (EID, **Figure 1B**) to estimate the binding strength between SFs and isoforms. Most splicing factor binding sites locate near the splicing sites [26], especially 200 bp on both sides of this site. Therefore, for isoforms with alternative splicing, we gathered 200 bp of sequences around their splicing sites into EID to find splicing factor binding sites.

Based on the PRD and EID, we could define the TF-isoform interaction strength and SF-isoform interaction strength. First, we downloaded TF motif models (PWM, position weight matrixes) from TRANSFAC [27] and JASPAR [28]. Then, for each TF, a hidden markov model based method called MAPPER [29] was used to identify its sites (TFBSs) in PRD. For each binding site, the MAPPER also outputs a binding score measuring the binding affinity. TFs with no hits on PRD were removed. After retrieving all putative binding sites and their binding scores, we defined the interaction strength between a TF 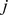 and a RNA isoform 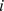 as 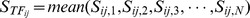. Where, 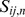 is the score of 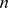-th binding site between 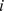-th isoform and *j*-th transcription factor; and 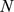 is the number of binding sites.

Unlike TFs, SFs lack a reliable PWM database available. Thus, we gathered 53 splicing factors that are related with bone marrow cancers and their binding motifs from the SpliceAid [30] repository. This database collects all the experimentally assessed motifs that are bound by splicing factors in humans by means of an exhaustive literature search. Motifs with positive scores facilitate exon definition as exonic splicing enhancers (ESEs) or intronic splicing silencers (ISSs) motifs, while motifs with negative scores facilitate intron definition as exonic splicing silencers (ESSs) and intronic splicing enhancers (ISE) motifs. The absolute values of these scores measure the levels of binding affinity. To control false positive rate, we filtered out motifs with absolute scores less than 5 and lengths longer than 15. Finally, 49 out of 53 splicing factors are selected (see Table 2). Since motifs of these splicing factors are degraded short pieces (6 to 10 base pairs on average), when retrieving all binding sites of these SFs using alignment tool Bowtie [31], we got numerous hits, most of which were false positives. To identify putative binding sites with high reliability, we downloaded base-wise conservation scores (phyloP) by comparing 45 vertebrate genomes with human genome from the UCSC Genome Browser website (http://genome.ucsc.edu/). We defined the conservation score of a hit as the average conservation score of the nucleotides it spanned. Hits with scores lower than 2 were deleted. Though conversion score is not directly related with interaction strength, a highly conserved binding site on an isoform does provide evidence of a strong interaction. We further averaged conservation scores of all hits of a splicing factor on an isoform to eliminate the effect of exon numbers. This average is defined as interaction strength between a SF and an isoform, 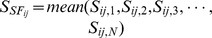, where, 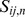 is the conservation score of 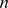-th binding site between 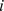-th isoform and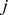-th splicing factor; 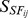 is the interaction strength between 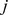-th splicing factor and 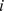-th isoform; 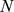
is the number of binding sites. Some factors, especially the hnRNP family, are due to alternative splicing, for example hnRNP H1, hnRNP H2 and hnRNP H3, which means they may have similar structure [32],motifs [33] and binding profiles. After we computed their interaction strength with isoforms according to our definition, the correlation between interaction strength vectors were high. To increase the robustness of our linear regression model, we averaged the binding strength and obtained four new factors, called hnRNP A1/A2 (average of hnRNP A1 and A2), hnRNP H1/H2/H3(average of hnRNP H1, H2 and H3), hnRNP C/C1/C2 (average of hnRNP C, C1 and C2) and hnRNP E1/E2 (average of E1 and E2).

**Table 2:** Names and description of splicing factors used in our model.

Splicing Factors	Gene Name	Description
**9G8**	SRSF7	Splicing factor arginine/serine-rich 7. The shuttling protein 9G8 binds TAP and can function as export factors.
**CUG-BP1**	CUGBP1	CUG triplet repeat RNA binding protein 1. CUGBP1 induces exon 5 inclusion in cTNT gene (PMID: 9563950), induces exon 11 exclusion in IR gene (PMID: 11528389), induces intron 2 retention in CIC-1 gene (PMID: 12150906).
**DAZAP1**	DAZAP1	DAZ associated protein 1.
**ETR-3**	CUGBP2	CUG triplet repeat RNA binding protein 2. ETR-3 induces exon 5 inclusion in cTNT gene (PMID: 11931771), induces exon 9 inclusion in CFTR gene (PMID: 15657417), promotes selectively the exclusion of Tau exon 2 (PMID: 16862542).
**hnRNP A1**	**HNRNPA1**	Heterogeneous nuclear ribonucleoprotein A1. hnRNP A1 carries bidirectional shuttling signals that serve for both nuclear localization and export (PMID: 8521471)
**hnRNP A2/B1**	HNRNPA2B1	Heterogeneous nuclear ribonucleoprotein A2/B1. hnRNP A2 is involved in cytoplasmic RNA transport (PMID: 11024030).
**hnRNP C**	HNRNPC	Heterogeneous nuclear ribonucleoprotein C. Tetramer composed of 3 copies of isoform C1 and 1 copy of isoform C2. hnRNP C proteins are restricted to the nucleus because they bear a nuclear retention sequence (NRS) (PMID: 8830767).
**hnRNP C1**	HNRNPC	Heterogeneous nuclear ribonucleoprotein C. Isoform C1 is due to Alternative Splicing.
**hnRNP C2**	HNRNPC	Heterogeneous nuclear ribonucleoprotein C. Isoform C2 is due to Alternative Splicing.
**hnRNP D**	HNRNPD	Heterogeneous nuclear ribonucleoprotein D.
**hnRNP D0**	HNRNPD	Heterogeneous nuclear ribonucleoprotein D. Isoform D0 is due to Alternative Splicing.
**hnRNP DL**	HNRNPDL	Heterogeneous nuclear ribonucleoprotein D-like.
**hnRNP E1**	PCBP1	Holy(rC) binding protein 1.
**hnRNP E2**	PCBP2	Holy(rC) binding protein 2.
**hnRNPF**	HNRNPF	Heterogeneous nuclear ribonucleoprotein F.
**hnRNPH1**	HNRNPH1	Heterogeneous nuclear ribonucleoprotein H1.
**hnRNPH2**	HNRNPH2	Heterogeneous nuclear ribonucleoprotein H2.
**hnRNPH3**	HNRNPH3	Heterogeneous nuclear ribonucleoprotein H3.
**hnRNP I(PTB)**	PTBP1	Polypyrimidine tract binding protein 1. In the context of CALCA gene, PTB enhances exon 4 inclusion (PMID: 9858533). nPTB functionally compensates for PTB and is up-regulated when PTB is removed (PMID:17679092).
**hnRNP J**	HNRNPK	Heterogeneous nuclear ribonucleoprotein K. isoform J is due to Alternative Splicing.
**hnRNP K**	HNRNPK	Heterogeneous nuclear ribonucleoprotein K. hnRNP K carries bidirectional shuttling signals that serve for both nuclear localization and export (PMID: 9218800).
**hnRNP M**	HNRNPM	Heterogeneous nuclear ribonucleoprotein M.
**hnRNP P(TLS)**	FUS	Fusion (involved in t (12, 16) in malignant liposarcoma).
**hnRNP Q**	SYNCRIP	Synaptotagmin binding cytoplasmic RNA interacting protein.
**hnRNP U**	HNRNPU	Heterogeneous nuclear ribonucleoprotein U (scaffold attachment factor A).
**HTra2alpha**	TRA2A	Transformer-2 alpha.
**HTra2beta1**	SRSF10	Splicing factor arginine/serine-rich 10.
**KSRP**	KHSRP	KH-type splicing regulatory protein.
**MBNL1**	MBNL1	Muscleblind-like. MBNL proteins can act as activators or repressors of splicing on different pre-mRNAs (PMID: 15257297). MBNLs are dsRNA binding factors that can bind CUG or CCUG repeats (PMID: 14722159).
**PSF**	SFPQ	Splicing factor proline/glutamine-rich (polypyrimidine tract binding protein associated).
**RBM25**	RBM25	RNA binding motif protein 25. RBM25 stimulated proapoptotic Bcl-X(s) isoform through weak 5′ss selection in EX2 (PMID: 18663000).
**RBM4**	RBM4	RNA binding motif protein 4. RBM4 induce exon inclusion of alpha-TM EX9a and EX2b (PMID: 16260624) and tau EX10 (PMID: 16777844).
**RBM5**	RBM5	RNA binding motif protein 5.
**Sam68**	KHDRBS1	KH domain containing RNA binding signal transduction associated 1.
**SC35**	SRSF2	Splicing factor arginine/serine-rich 2. SC35 accelerates transcriptional elongation (co-transcriptional splicing) (PMID: 18641664).
**SF1**	SF1	Splicing factor 1. Gomafu lncRNA UACUAAC repeats bind to mouse SF1 with a higher affinity than the mammalian branch point consensus regulating splicing efficiency by changing the splicing factors nuclear level (PMID: 21463453)
**SF2**	SRSF1	Splicing factor arginine/serine-rich 1 (splicing factor 2, alternate splicing factor). The shuttling protein SF2/ASF binds TAP and can function as export factors (18364396).
**SRp20**	SRSF3	Splicing factor arginine/serine-rich 3. The shuttling protein SRp20 binds TAP and can function as export factors (18364396).
**SRp30c**	SRSF9	Splicing factor arginine/serine-rich 9.
**SRp38**	FUSIP1	FUS interacting protein (serine/arginine-rich). Dephosphorylation converts SRp38 to a splicing repressor (PMID: 12419250). SRp38 functions as a general splicing repressor when dephosphorylated, but when phosphorylated it functions as a sequence-specific splicing activator (PMID: 18794844).
**SRp40**	SRSF5	Splicing factor arginine/serine-rich 5.
**SRp54**	SRSF11	Splicing factor, arginine/serine-rich 11.
**SRp55**	SRSF6	Splicing factor arginine/serine-rich 6.
**SRp75**	SRSF4	Splicing factor arginine/serine-rich 4.
**TDP43**	TARDBP	TAR DNA binding protein. It can act as transcriptional repressor (21252238).
**TIA-1**	TIA1	Cytotoxic granule-associated RNA binding protein.
**TIAL1**	TIAL1	TIA1 cytotoxic granule-associated RNA binding protein-like 1.
**YB-1**	YBX1	Y box binding protein 1.
**ZRANB2**	ZRANB2	Zinc finger, RAN-binding domain containing 2. ZRANB2 (ZNF265) is an SR-like protein that induce exclusion of EX2 and EX3 from the Tra2beta1 pre-mRNA in HEK293 cell (PMID: 11448987).

This table contains 22 splicing factors which are selected to predict the expression levels of differentially expressed isoforms. This table lists their names and some related references. Most of these details are from SpliceAid.

Integrating all these binding strength, the results were two interaction strength matrices with rows corresponding to isoforms and columns corresponding to TFs or SFs. These were bound together to build linear regression model in equation (1) to fit isoform expression levels (**Figure 1C**).

### Model Selection

The dependent variable (isoform expression) and explanatory variables (interaction strength value of factors) in the linear regression model in (1) were all normalized. Many methods such as ordinary least squares (OLS), ridge regression with a L2 norm penalization [34], LASSO with a L1 norm term [35], and Least-angle regression (LARS), can be adopted for optimization and selection of linear model. However, when the number of explanatory variables is higher than number of observations, it is more critical to balance regression accuracy and interpretation capability. Unfortunately, the OLS method incurs both regression accuracy and interpretation ability problems. Ridge regression fails to achieve a parsimonious set of predictors, which may lead to stable regression accuracy but a poor interpretation of model. LASSO, in which a L1 norm penalization term is employed, helps to improve both issues. However, it adopts quadratic programming techniques to solve a constrained optimization problem, which may limit its application when the sample size is small with respect to the number of explanatory variables. Therefore, in this paper, we adopted a promising model selection method called LARS [12], which can balance the accuracy and interpretation capability better, to selected TF and SF factors that regulated each group of co-expressed isoforms. The LARS algorithm is summarized in **Table 3**.

**Table 3:** Pseudocode of LARS algorithm.

LARS algorithm
Data: Normalized expression levels of co-expressed isoforms 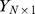 , normalized interaction strength matrix *X_N_*_×*P*_
Output: Regression coefficients *α_P_*_×1_
All coefficients 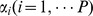 equal to zero;
Active set 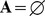 ;
Find predictor 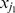 most correlated with *Y_N_*_×1_;
Let direction 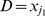 ;
Repeat
Adjust the coefficient in the direction 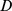 at the highest step possible until some other explanatory variable 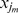 has the same absolute correlation residual 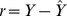 ;
Put 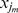 in 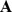 ;
Let 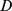 in the direction that is equiangular with 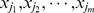
Until 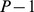 variables have entered the active set

### Biological Significance of these Regulatory Networks

At the last step of the flowchart in **Figure 1**, a transcription and splicing network (TSN) was constructed with a group of co-expressed isoforms and the selected TFs and SFs using the method described above. To validate the biological significance of these regulatory networks we conducted a comprehensive functional enrichment analysis.

First, we studied the enrichment of these inferred regulatory networks in the KEGG pathway [36]. The KEGG database is a collection of manually drawn pathway maps representing exiting knowledge of the molecular interaction and reaction networks. We mapped all items (isoforms, TFs and SFs) in TSNs to their ENTREZ ID and gathered all KEGG pathways from http://www.genome.jp/kegg/. The enrichment of a network in a pathway was evaluated using Fisher’s exact test.

We also used the Gene Ontology (GO) [37] database for enrichment analysis. This project defines GO terms and structures GO ontology as a directed acyclic graph through which each term has relationships to one or more other terms in the same domain. We downloaded GO biological processes through package GOSim [38] for R language. Considering the size of our regulatory networks, we filtered out GO biological processes involving more than 100 genes or fewer than 10 genes. Fisher’s exact test was performed using enrichment analysis function (GOenrichment) in the GOSim package.

## Results

To demonstrate how our method works, we first looked at results of linear regression. After regression and model selection, 31 networks were obtained. Three co-expressed groups that did not fit the linear model well were removed. **Table 1** lists the gene names of each co-expressed group and the corresponding TFs and SFs identified by our method. Here we choose Network 1 as an example for further explanation (**Table 4**). In this network, there are 12 target co-expressed isoforms. For convenience, we used gene names instead of isoform names. The LARS algorithm identified 5 transcription factors and 5 splicing factors that may regulate their expression. The adjusted coefficient of determination (adjusted R squared) is 0.991, which means expression of these co-expressed isoforms was well fitted by their interaction with the selected factors. Coefficients in regression are indicators of contribution from corresponding factors. Factors with higher coefficients, such as MBNL1, PSF, Sam68 and SRp38, predominate in influencing expression of this group of isoforms. On the other hand, factors with lower coefficients, such as FOX factors and Blimp-1, contribute less to determining expression of their targets. Some of these coefficients are negative, which means these factors may inhibit these isoforms’ expression. Details of the regression coefficients are also listed (**Table 4**). Many factors may be involved in the expression of these isoforms. Here, LARS only selected the most representative ones that not only could regulate the expression of these isoforms linearly but also did not suffer from an over-fitting problem.

**Table 4:** Target genes and corresponding factors in networks 1.

TFs	Coefficients	SFs	Coefficients	Target genes	Adjusted R
**Blimp-1**	0.0341	Sam68	1.082	GNB1 PSPC1ATP5J SETD3	
**Sp1**	−0.303	SF2	0.106	GPATCH4 HNRNPD	
**Fox factors**	0.023	SRp38	−0.6486	MRPL51 RBBP7	0.991
**FOXP1a**	−0.387	MBNL1	−1.106	BRD4 CALM3	
**STAT5B**	−0.012	PSF	−0.681	ODC1 ANP32C	

This table lists the target genes and factors that regulate them. The regression coefficients are listed on the right side.

We also checked all the differentially expression isoforms using Ingenuity Pathway Analysis (IPA, http://www.ingenuity.com/index.html). In all 14 genes (FLT3, KIT, RPL13A, RPL3, RPL6, RPL7, RPS14, RPS15A, RPS16, RPS19, RPS20, RPS4X, RPS6 and RUNX1) had been reported in MDS. Our isoform analysis asserted that they all experienced alternative splicing. However when we used traditional gene array analysis (7 MDS cases, 7 controls downloaded from Gene Expression Omnibus [Gene Expression Omnibus number: GSE16236]), only 2 were recognized as being differentially expressed. This result demonstrated that using only gene expression analysis is not sufficient. Isoform and splicing analysis provides more information about gene profiles and their regulatory mechanism.

### MDS-related Factors

We then analyzed the transcription and splicing factors detected by our method. Some of these factors have already been linked with MDS or cancers in the literature, while others, such as SRSF11, may be candidate factors that need further validation.

#### EGR1

The early growth response gene EGR1 appeared in 58% (18 of 31) of our networks. This factor plays important roles in the regulation of cell growth, differentiation, and survival and has been confirmed as a candidate tumor suppressor gene within the commonly deleted segment of 5 q in MDS. However, accumulating evidence now indicates that it can act a tumor promoter in some cancer, such as prostate [39]. None of our MDS samples are del(5 q), the EGR1 was overexpressed in all disease samples compared to controls,. This suggests that EGR1 plays a significant role in the development of MDS and that its function in non-del(5 q) MDS needs further study.

#### STAT family

Like EGR1, the STAT family is also involved in cell growth, differentiation, and survival and plays a role as candidate regulator in almost one third of our networks. This group of factors is typically oncogenic through the constitutive activation of tyrosine kinase. The most canonical pathway for the STAT family is FLT3 signaling in hematopoietic progenitor cells. This relationship appeared in our network 18. In this network, two STATs were predicted to regulate the expression of FLT3. Though FLT3 positively regulates the tyrosine phosphorylation of STAT proteins in the FLT3 signaling pathway, STATs are still on the card to form signaling by regulating the expression level of FLT3 through an auto-regulatory loop. He *et al.* [40] discussed a positive auto-regulatory loop in the Jak-STAT pathway. STAT1/3 in this loop tends to induce the expression of various components of the Jak-STAT pathway to strengthen the signaling. Over activation of FLT3 via the ITD mutation is related to the pathogenesis of AML/MDS and is an adverse prognosis marker.

#### SRSF11

Recent studies reported some recurrent mutated splicing factors in MDS [16], [41], [42]. These mutated genes, including U2AF35, ZRSR2, SRSF2, SF3B1, SF3A1, SF1, U2AF65 and PRPF40B were involved in multiple components of the RNA splicing mechanism. However, none of these splicing factors were recurrently mutated in our MDS cases.

**Figure 2:**
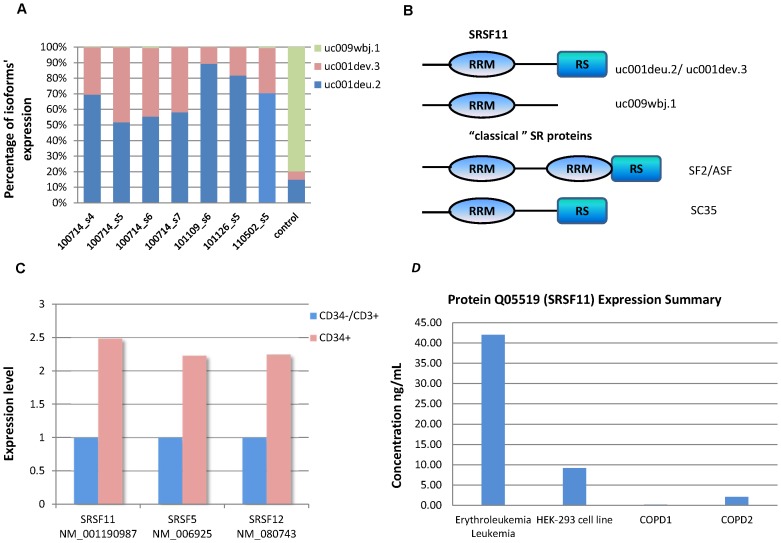
Expression ratio of SRSF11’s three isoforms (A), motifs in SRSF11’s isoforms and classical SR proteins (B), RT-PCR results (C) and protein Expression of SRSF11. (A). Expression ratio of SRSF11’s three isoforms in seven disease sample and control: uc009wbj.1 (light green), uc001deu.2 (light blue) and uc001.dev.3 (light red). They have almost the same total expression levels but very different ratios in MDS (four ) and control (average of five controls), which means the splicing patterns of SRSF11 are switched. (B). This figure demonstrates motifs in SRSF11’s isoforms and classical SR proteins. Different motifs have different bio-function. (C). Three isoforms that are over-expressed in our disease samples are picked up for RT-PCR validation. They are isoforms of three splicing factor, one isoform (uc001deu.2, refseq ID: NM_001190987) of SRSF11, one isoform (uc001xlp.3, refseq ID: NM_006925) of SRSF5 and one isoform (uc003jun.2, refseq ID:NM_080743) of SRSF12. Validation demonstrated that their expression levels in MDS disease are higher than in control. (D). Isofrom uc001deu.2 is translated into protein Q05519 and Q05519 is highly expressed in blood disease according to the Model Organism Protein Expression Database (MOPED); COPD: Chronic obstructive pulmonary disease.

We downloaded splicing factors from RBPDB [43] and checked for alternative splicing. Among these 40 splicing factors, SRSF11 showed isoform switching in 7 of 20 MDS samples (**Figure 2A**), including 4 RAEBs used to build our model, one RCMD, one AML with MDS and one MDS for which the subtype is unknown. According to the annotation information from the UCSC database, the SRSF11 gene has eight isoforms, three of which (uc001deu.2, uc001dev.3, uc009wbj.1) are highly expressed in our samples. Isoform uc001deu.2 and uc001dev.3 have evidence at the protein level (called p54, Uniprot ID Q05519), while the protein of uc009wbj.1 has not been found yet. Although the total expression levels of these three isoforms were almost the same in MDS and control samples, 2 isoforms (uc001deu.2, uc001dev.3) were highly expressed in MDS samples, whereas the uc009wbj.1 isoform was highly expressed in control (average expression values). The shorter amino acid chain from uc009wbj.1 only contained the RRM (RNA recognition motif), while p54 not only contained RRM at the N-terminal but also had a C-terminal RS domain. This domain promotes protein-protein interactions to facilitate recruitment of the spliceosome [7], [44], [45] (**Figure 2B**). These two kinds of splicing factors (with or without SR domain) exhibit two different functions. One is RS-domain dependent (recruiting function) and the other is RS-domain independent (antagonist function) [46]. Hence, we hypothesized that, in MDS cases, the recruiting function of SRSF11 was enhanced and the antagonizing splicing inhibitors was weakened. However, we needed further data to evaluate the effect of this splicing switch.

To validate the abnormal expression of some isoforms, reverse transcription polymerase chain reaction (RT-PCR) techniques (**File S1**) were used to measure the expression level of three isoforms from three SR proteins, including one isoform (uc001deu.2, refseq ID: NM_001190987) of SRSF11, one isoform (uc001xlp.3, refseq ID: NM_006925) of SRSF5 and one isoform (uc003jun.2, refseq ID: NM_080743) of SRSF12. CD34+ and CD34- cells were sorted and CD34−/CD3+ cells were further separated with CD34 and CD3 magnetic beads (Miltenyi Biotec, Auburn, CA). Total cellular RNA was extracted and cDNA was synthesized as described in [47]. We chose primer 5′-GCCTGGGCTGGAGGACAGAGA-3′ and 5′-TGCTCGGGTTCTCGCTCTTGATTG-3′ for SRSF11 (NM_001190987),5′- TGCGTCAGTTGTGGAGTGGCG-3′ and 5′-CGGCTAGTACTTCCGGACGGGG-3′ for SRSF5 (NM_006925), 5′-CGGGAGACGGAGCGAGTCCA-3′ and 5′- TCCTCAGGCCTGG TGGCGTC-3′ for SRSF12 (NM_080743), 5′-TTCGGAACTGA GGCCATGAT-3′ and 5′-TTTCGCTCTGGTCCGTCTTG-3′ for human 18SrRNA as the house keeping gene. The amplification process was conducted on the LightCycler with FastStart DNA Master SYBR®Green (Roche Applied Science, Indianapolis, IN). All three isoforms, tended to be highly expressed in MDS (**Figure 2C**), consistent with the observation that increased expression of SR proteins usually correlates with cancer progression [48].

We also downloaded the protein expression profile of SRSF11 from the Model Organism Protein Expression Database (MOPED). We found that SRSF11 protein is highly expressed in hematologic diseases (**Figure 2D**). It appears that the higher expression of SRSF11 protein is due to the higher expression of uc001deu.2 and uc001dev.3.

### Enrichment Analysis

To evaluate the biological function of these 31 networks, we comprehensively analyzed their enrichment in KEGG pathways and GO biological process terms using the Fisher-exact test. Twenty (64.5%) of 31 networks were enriched in at least one KEGG pathway with an FDR-corrected q-value<0.05. **Table 5** lists the MDS-related networks. The most enriched pathway is herpes simplex infection in which splicing factors are extensively involved. The second most enriched pathway is pathway in cancer. This is a very general pathway including many diseases, including AML, due to its important role in proliferation. There were also two networks (NT18 and NT20) enriched in the acute myeloid leukemia pathway, the PPAR signaling pathway and the Jak-STAT signaling pathway. Though these pathways are reported with AML, our RAEB subtype which has high risk of transforming to AML should a have similar gene profiles with AML.

**Table 5:** Results of enrichment analysis using KEGG database.

No.	Pathway	Regulatory Network
**1**	Herpes simplex infection	NT2, NT6, NT7, NT8, NT9, NT10, NT11, NT12, NT13, NT15, NT16, NT18, NT20, NT22, NT25, NT31
**2**	Pathways in cancer	NT8, NT9, NT14, NT15, NT18, NT20
**3**	Chronic myeloid leukemia	NT9, NT14, NT18, NT22
**4**	Maturity onset diabetes of the young	NT15, NT25, NT32
**5**	Acute myeloid leukemia	NT18, NT20
**6**	PPAR signaling pathway	NT15, NT20
**7**	Pertussis	NT8, NT22
**8**	Transcriptional mis-regulation in cancer	NT15
**9**	Jak-STAT pathway	NT18

This table lists top enriched KEGG pathways and corresponding networks number.

These networks were also enriched in 42 different GO biological processes and 21 (68%) were enriched in at least one process (Pvalue <1e-4). **Table 6** lists three selected networks and their corresponding biological processes. Most of these biological processes are related with splicing, including mRNA 5′-splice site recognition, regulation of RNA splicing, and mRNA 3′-end processing.

**Table 6:** Results of enrichment analysis using GO database.

Network	Biological Process	P value
**6**	mRNA splicing, via spliceosome	5.8e-05
	leading edge cell differentiation	2.2e-05
	ERK5 cascade	3.9e-6
**10**	mRNA splice site selection	8.8e-05
**11**	regulation of RNA splicing	1.2e-05
	positive regulation of mRNA splicing, via spliceosome	5.5e-05
	positive regulation of transcription initiation from RNA polymerase II promoter	7.4e-06
	positive regulation of neural precursor cell proliferation	3.8e-05
**15**	mRNA 5′-splice site recognition	2.9e-05
	regulation of muscle cell differentiation	1.3e-07
**16**	transcription from RNA polymerase II promoter	3.1e-05
	termination of RNA polymerase II transcription	3.8e-05
	mRNA 3′-end processing	1.0e-07
	multi-organism reproductive process	4.7e-06

Three networks enriched in some GO biological processes. This table lists the details and the P values.

## Discussion

From transcription to translation, gene expression is modulated by many factors. Traditional predictive models of gene expression only consider the transcription. In this study, we proposed a systematic approach to recognize putative regulatory factors regulating co-expressed isoforms that were differentially expressed in disease. In case of MDS, the most recurrent transcription factors involved in regulating abnormally expressed genes were NKX2-5 and Egr-1. NKX2-5 is a master transcription factor. EGR1 is a candidate tumor suppressor gene within the commonly deleted segment of 5 q and has been claimed to play a role in murine leukemogenesis and development of AML/MDS characterized by abnormalities of chromosome 5. Its overexpression in our MDS cases indicates it may also act as tumor promoter as in prostate cancer. Additionally, we found some putative MDS-associated splicing factors, e.g. SF2 and SRSF11. They were highly related with developmental pathways that were deregulated in MDS cases. Previous reports confirm that SF2 is an oncogene and overexpression of SF2 may cause some tumor suppressors to lose function [49]. Our MDS samples verified its overexpression. We also detected a significant splicing switch of factor SFRS11. The ratio of the isoforms produced by the alternative splicing of SFRS11’s pre-mRNA is significantly different in controls and MDS samples. This provided evidence that aberrant expression and regulation of splicing factors may result in the deregulation of splicing in diseases. Overall, our method is a good choice to detect these disease-related factors.

**Figure 3:**
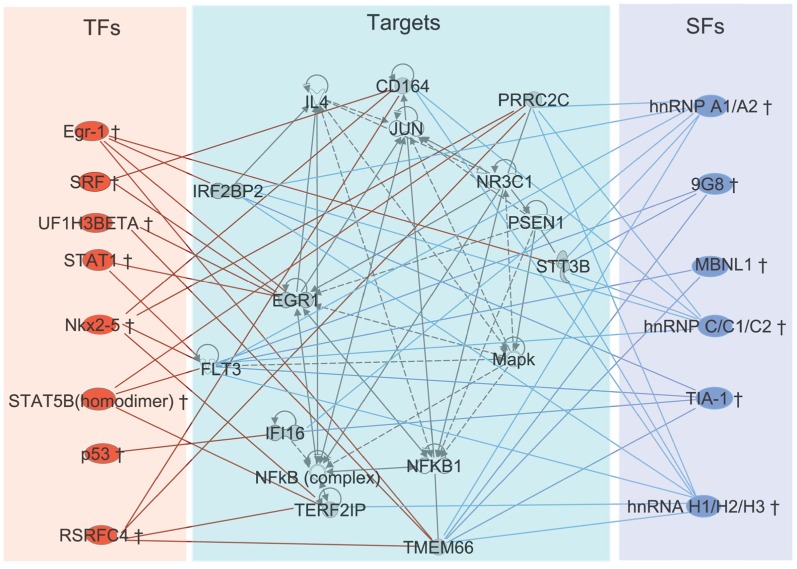
Connected regulatory network for network 18. Connected regulatory network for network 18. Red nodes are transcription factors, blue nodes are splicing factors and gray nodes in the middle are targets. The connection between targets is from IPA and the connection between factors and targets are from our interaction strength matrix.

In addition, this study offered a method to construct transcription and splicing networks. Taking network 18 (**Table 1**) as an example. We first input the target genes (DEIs) into the IPA system, and found that these genes are enriched in hematological system development and function, gene expression and cellular development networks. In order to look into the details, only a sub graph (dark nodes in the middle of **Figure 3**) was displayed. Then we added TFs and SFs to demonstrated regulatory relation. All these edges between target genes and factors (TFs and SFs) were determined based on the interaction strength matrix. Although the connection between factors and target genes were determined by our algorithm instead of experiments, some of them were supported by literatures. For example p53 is one of the most important tumor suppressors. In our network, it was connected with the IFI16 (interferon-inducible myeloid differentiation transcriptional activator), which was consistent with the results in [50]. Those previous experiments indicated that p53 could up-regulate IFI16, and that functional interactions between IFI16 protein and p53 contributed to cellular senescence. The relationship between the FLT and the STAT family was supported by the FLT signaling pathway. Though FLT3 is upstream of the STAT family, it is very likely that a regulatory loop like in the Jak-STAT pathway [40] exists. In myeloid progenitor cells, Egr-1 bound to the Egr-1 promoter [51]. The regulation of STAT1 to EGR1 has also been postulated [52]. However, some novel connections that have not yet been reported, and their reliability needs further validation.

We suggested that splicing factor SRSF11 might function differently in MDS patients and controls. To comprehensively examine its target genes, we screened the whole genome using its reported motif and found a number of putative binding sites. Conservation scores for each binding site were also computed to control the false positive rate. Only one-third of hits with the highest conservation scores were kept for further analysis. Finally, we obtained 1148 conserved binding sites, corresponding to 158 genes. They were all putative targets of SRSF11. All these genes were analyzed using IPA software. The associated network with the highest scores were those involved in cellular development, cellular growth and proliferation and cell morphology. A total of 29 genes were associated with cancers, and 12 were associated with hematological disease, including the MTOR gene which is in the Akt/mTOR pathway and is critical for cell survival and proliferation in high-risk MDS patients [53]. Since the Akt/mTOR has been advised as a therapeutic target for treating MDS, our study suggested that its abnormality might be related with the splicing switch of SRSF11.

## Supporting Information

File S1 **RT-PCR.** A portable document format (pdf) file contains details of RT-PCR.
(PDF)

## References

[1] Beer MA, Tavazoie S (2004) Predicting gene expression from sequence. Cell 117: 185–198. 15084257 10.1016/s0092-8674(04)00304-6

[2] Conlon EM, Liu XS, Lieb JD, Liu JS (2003) Integrating regulatory motif discovery and genome-wide expression analysis. Proc Natl Acad Sci U S A 100: 3339–3344. 12626739 10.1073/pnas.0630591100 PMC152294

[3] Matlin AJ, Clark F, Smith CWJ (2005) Understanding alternative splicing: towards a cellular code. Nat Rev Mol Cell Biol 6: 386–398. 15956978 10.1038/nrm1645

[4] Tang ZZ, Sharma S, Zheng S, Chawla G, Nikolic J, et al (2011) Regulation of the mutually exclusive exons 8a and 8 in the CaV1.2 calcium channel transcript by polypyrimidine tract-binding protein. J Biol Chem 286: 10007–10016. 21282112 10.1074/jbc.M110.208116 PMC3060452

[5] Chaudhury A, Chander P, Howe PH (2010) Heterogeneous nuclear ribonucleoproteins (hnRNPs) in cellular processes: Focus on hnRNP E1’s multifunctional regulatory roles. RNA 16: 1449–1462. 20584894 10.1261/rna.2254110 PMC2905745

[6] Dreyfuss G, Kim VN, Kataoka N (2002) Messenger-RNA-binding proteins and the messages they carry. Nat Rev Mol Cell Biol 3: 195–205. 11994740 10.1038/nrm760

[7] Long JC, Caceres JF (2009) The SR protein family of splicing factors: master regulators of gene expression. Biochem J 417: 15–27. 19061484 10.1042/BJ20081501

[8] Screaton GR, Cáceres JF, Mayeda A, Bell MV, Plebanski M, et al (1995) Identification and characterization of three members of the human SR family of pre-mRNA splicing factors. EMBO J 14: 4336–4349. 7556075 10.1002/j.1460-2075.1995.tb00108.x PMC394518

[9] Zahler A, Neugebauer K, Lane W, Roth M (1993) Distinct functions of SR proteins in alternative pre-mRNA splicing. Science 260: 219–222. 8385799 10.1126/science.8385799

[10] Wang J, Manley JL (1995) Overexpression of the SR proteins ASF/SF2 and SC35 influences alternative splicing in vivo in diverse ways. RNA 1: 335–346. 7489505 PMC1369086

[11] Trapnell C, Roberts A, Goff L, Pertea G, Kim D, et al (2012) Differential gene and transcript expression analysis of RNA-seq experiments with TopHat and Cufflinks. Nat Protoc 7: 562–578. 22383036 10.1038/nprot.2012.016 PMC3334321

[12] Efron B, Hastie T, Johnstone I, Tibshirani R (2004) Least Angle Regression. The Annals of Statistics 32: 407–451.

[13] Harris NL, Jaffe ES, Diebold J, Flandrin G, Muller-Hermelink HK, et al (2000) The World Health Organization classification of neoplasms of the hematopoietic and lymphoid tissues: report of the Clinical Advisory Committee meeting–Airlie House, Virginia, November, 1997. Hematol J 1: 53–66. 11920170 10.1038/sj.thj.6200013

[14] Estey E, Thall P, Beran M, Kantarjian H, Pierce S, et al (1997) Effect of diagnosis (refractory anemia with excess blasts, refractory anemia with excess blasts in transformation, or acute myeloid leukemia [AML]) on outcome of AML-type chemotherapy. Blood 90: 2969–2977. 9376577

[15] Bejar R, Stevenson K, Abdel-Wahab O, Galili N, Nilsson B, et al (2011) Clinical effect of point mutations in myelodysplastic syndromes. N Engl J Med 364: 2496–2506. 21714648 10.1056/NEJMoa1013343 PMC3159042

[16] Graubert TA, Shen D, Ding L, Okeyo-Owuor T, Lunn CL, et al (2012) Recurrent mutations in the U2AF1 splicing factor in myelodysplastic syndromes. Nat Genet 44: 53–57. 10.1038/ng.1031 PMC3247063 22158538

[17] Trapnell C, Hendrickson DG, Sauvageau M, Goff L, Rinn JL, et al (2013) Differential analysis of gene regulation at transcript resolution with RNA-seq. Nat Biotechnol 31: 46–53. 23222703 10.1038/nbt.2450 PMC3869392

[18] Eisen MB, Spellman PT, Brown PO, Botstein D (1998) Cluster analysis and display of genome-wide expression patterns. Proc Natl Acad Sci U S A 95: 14863–14868. 9843981 10.1073/pnas.95.25.14863 PMC24541

[19] Spellman PT, Rubin GM (2002) Evidence for large domains of similarly expressed genes in the Drosophila genome. J Biol 1: 5. 12144710 10.1186/1475-4924-1-5 PMC117248

[20] Stuart JM, Segal E, Koller D, Kim SK (2003) A gene-coexpression network for global discovery of conserved genetic modules. Science 302: 249–255. 12934013 10.1126/science.1087447

[21] Allocco DJ, Kohane IS, Butte AJ (2004) Quantifying the relationship between co-expression, co-regulation and gene function. BMC Bioinformatics 5: 18. 15053845 10.1186/1471-2105-5-18 PMC375525

[22] Caron H, van Schaik B, van der Mee M, Baas F, Riggins G, et al (2001) The human transcriptome map: clustering of highly expressed genes in chromosomal domains. Science 291: 1289–1292. 11181992 10.1126/science.1056794

[23] Gabut M, Samavarchi-Tehrani P, Wang X, Slobodeniuc V, O’Hanlon D, et al (2011) An alternative splicing switch regulates embryonic stem cell pluripotency and reprogramming. Cell 147: 132–146. 21924763 10.1016/j.cell.2011.08.023

[24] Bussemaker HJ, Li H, Siggia ED (2001) Regulatory element detection using correlation with expression. Nat Genet 27: 167–171. 11175784 10.1038/84792

[25] Garg A, Lohmueller JJ, Silver PA, Armel TZ (2012) Engineering synthetic TAL effectors with orthogonal target sites. Nucleic Acids Res 40: 7584–7595. 22581776 10.1093/nar/gks404 PMC3424557

[26] Majewski J, Ott J (2002) Distribution and characterization of regulatory elements in the human genome. Genome Res 12: 1827–1836. 12466286 10.1101/gr.606402 PMC187578

[27] Matys V, Fricke E, Geffers R, Gössling E, Haubrock M, et al (2003) TRANSFAC: transcriptional regulation, from patterns to profiles. Nucleic Acids Res 31: 374–378. 12520026 10.1093/nar/gkg108 PMC165555

[28] Sandelin A, Alkema W, Engstrom P, Wasserman WW, Lenhard B (2004) JASPAR: an open-access database for eukaryotic transcription factor binding profiles. Nucleic acids research 32: D91–D94. 14681366 10.1093/nar/gkh012 PMC308747

[29] Riva A (2012) The MAPPER2 Database: a multi-genome catalog of putative transcription factor binding sites. Nucleic Acids Res 40: D155–D161. 22121218 10.1093/nar/gkr1080 PMC3245066

[30] Piva F, Giulietti M, Burini AB, Principato G (2012) SpliceAid 2: a database of human splicing factors expression data and RNA target motifs. Hum Mutat 33: 81–85. 21922594 10.1002/humu.21609

[31] Langmead B, Trapnell C, Pop M, Salzberg SL (2009) Ultrafast and memory-efficient alignment of short DNA sequences to the human genome. Genome Biol 10: R25. 19261174 10.1186/gb-2009-10-3-r25 PMC2690996

[32] Lorkovic ZJ (2012) RNA Binding Proteins. Austin: Landes Bioscience.

[33] Piva F, Giulietti M, Burini AB, Principato G (2012) SpliceAid 2: a database of human splicing factors expression data and RNA target motifs. Hum Mutat 33: 81–85. 21922594 10.1002/humu.21609

[34] Hoerl AE, Kennard RW (1970) Ridge Regression: Biased Estimation for Nonorthogonal Problems. Technometrics 12: 55–67.

[35] Tibshirani R (1996) Regression Shrinkage and Selection via the Lasso. Journal of the Royal Statistical Society Series B (Methodological) 58: 267–288.

[36] Ogata H, Goto S, Sato K, Fujibuchi W, Bono H, et al (1999) KEGG: Kyoto Encyclopedia of Genes and Genomes. Nucleic Acids Res 27: 29–34. 9847135 10.1093/nar/27.1.29 PMC148090

[37] Ashburner M, Ball CA, Blake JA, Botstein D, Butler H, et al (2000) Gene ontology: tool for the unification of biology. The Gene Ontology Consortium. Nat Genet 25: 25–29. 10802651 10.1038/75556 PMC3037419

[38] Froehlich H (2012) GOSim: Computation of functional similarities between GO terms and gene products; GO enrichment analysis.

[39] Yang SZ, Eltoum IA, Abdulkadir SA (2006) Enhanced EGR1 activity promotes the growth of prostate cancer cells in an androgen-depleted environment. J Cell Biochem 97: 1292–1299. 16552752 10.1002/jcb.20736

[40] He F, Ge W, Martinowich K, Becker-Catania S, Coskun V, et al (2005) A positive autoregulatory loop of Jak-STAT signaling controls the onset of astrogliogenesis. Nat Neurosci 8: 616–625. 15852015 10.1038/nn1440 PMC4222251

[41] Papaemmanuil E, Cazzola M, Boultwood J, Malcovati L, Vyas P, et al (2011) Somatic SF3B1 mutation in myelodysplasia with ring sideroblasts. N Engl J Med 365: 1384–1395. 21995386 10.1056/NEJMoa1103283 PMC3322589

[42] Yoshida K, Sanada M, Shiraishi Y, Nowak D, Nagata Y, et al (2011) Frequent pathway mutations of splicing machinery in myelodysplasia. Nature 478: 64–69. 21909114 10.1038/nature10496

[43] Cook KB, Kazan H, Zuberi K, Morris Q, Hughes TR (2011) RBPDB: a database of RNA-binding specificities. Nucleic Acids Res 39: D301–D308. 21036867 10.1093/nar/gkq1069 PMC3013675

[44] Shen H, Green MR (2004) A pathway of sequential arginine-serine-rich domain-splicing signal interactions during mammalian spliceosome assembly. Mol Cell 16: 363–373. 15525510 10.1016/j.molcel.2004.10.021

[45] Shen H, Kan JLC, Green MR (2004) Arginine-serine-rich domains bound at splicing enhancers contact the branchpoint to promote prespliceosome assembly. Mol Cell 13: 367–376. 14967144 10.1016/s1097-2765(04)00025-5

[46] Cartegni L, Chew SL, Krainer AR (2002) Listening to silence and understanding nonsense: exonic mutations that affect splicing. Nat Rev Genet 3: 285–298. 11967553 10.1038/nrg775

[47] Wen J, Feng Y, Bjorklund CC, Wang M, Orlowski RZ, et al (2011) Luteinizing Hormone-Releasing Hormone (LHRH)-I antagonist cetrorelix inhibits myeloma cell growth in vitro and in vivo. Mol Cancer Ther 10: 148–158. 21062912 10.1158/1535-7163.MCT-10-0829

[48] Fischer D-C, Noack K, Runnebaum IB, Watermann DO, Kieback DG, et al (2004) Expression of splicing factors in human ovarian cancer. Oncol Rep 11: 1085–1090. 15069551

[49] Karni R, de Stanchina E, Lowe SW, Sinha R, Mu D, et al (2007) The gene encoding the splicing factor SF2/ASF is a proto-oncogene. Nat Struct Mol Biol 14: 185–193. 17310252 10.1038/nsmb1209 PMC4595851

[50] Song LL, Alimirah F, Panchanathan R, Xin H, Choubey D (2008) Expression of an IFN-inducible cellular senescence gene, IFI16, is up-regulated by p53. Mol Cancer Res 6: 1732–1741. 18974396 10.1158/1541-7786.MCR-08-0208

[51] Herdegen T, Leah JD (1998) Inducible and constitutive transcription factors in the mammalian nervous system: control of gene expression by Jun, Fos and Krox, and CREB/ATF proteins. Brain Res Brain Res Rev 28: 370–490. 9858769 10.1016/s0165-0173(98)00018-6

[52] Ingram JL, Antao-Menezes A, Mangum JB, Lyght O, Lee PJ, et al (2006) Opposing actions of Stat1 and Stat6 on IL-13-induced up-regulation of early growth response-1 and platelet-derived growth factor ligands in pulmonary fibroblasts. J Immunol 177: 4141–4148. 16951379 10.4049/jimmunol.177.6.4141

[53] Follo MY, Mongiorgi S, Bosi C, Cappellini A, Finelli C, et al (2007) The Akt/mammalian target of rapamycin signal transduction pathway is activated in high-risk myelodysplastic syndromes and influences cell survival and proliferation. Cancer Res 67: 4287–4294. 17483341 10.1158/0008-5472.CAN-06-4409

