# Supplementary material for: Discovering Transcription and Splicing Networks in Myelodysplastic Syndromes
Source: PLoS One. 2013 Nov 14;8(11):e79118. doi: 10.1371/journal.pone.0079118 (PMC3828332; doi:10.1371/journal.pone.0079118)
Supplement: File S1 — RT-PCR. A portable document format (pdf) file contains details of RT-PCR. (PDF) [file pone.0079118.s001.pdf]

Quantitative RT-PCR

RNA were prepared with were directly lysed in Nugen lysis buffer (Nugen, San Carlos, CA), then transcribed into cDNA with the WT-Ovation RNA Amplification System (NuGEN) and analyzed according with user's guide. Quantitative RT-PCR analysis of the gene expression was then performed using RT2-SYBR®Green PCR Master Mix (SABioscience, Qiagen) with 40 cycles of 15 seconds at 95ºC and 1 minute at 58 ºC on an ABI 7500 Real-Time PCR System (Applied Biosystems, Foster City, CA). Fluorescence data were collected at 58 ºC after each cycle. After the final cycle, melting curve analysis of all samples was conducted within the range of 58 ºC -95 ºC. The specificity of the PCR products of each PCR reaction was verified by the targeted product size by gel electrophoresis. The threshold cycle and 2-ΔΔ t method were used for calculating the relative amount of the target RNA using the average of 5 house-keeping genes as internal control, according to user's manual. The experiments were repeated in triplicate.
